# Statewide surveillance of tick-borne pathogens in ticks collected in Delaware using novel multiplex PCR assays

**DOI:** 10.1016/j.ijppaw.2025.101058

**Published:** 2025-03-21

**Authors:** Michael H. Buoni, Ashley C. Kennedy, Virginia Hughes, Esther Biswas-Fiss

**Affiliations:** a University of Delaware, USA; b Delaware Technical Community College, USA; c Delaware Department of Natural Resources and Environmental Control, USA

**Keywords:** Multiplex PCR assay, *Borrelia burgdorferi*, *Babesia microti*, *Anaplasma phagocytophilum*, *Ehrlichia chaffeensis and Ehrlichia ewingii*, *Delaware*

## Abstract

Tick-borne pathogens are responsible for most vector-borne human diseases in the United States. With the growing recognition of tick-borne diseases and the expanding range of ticks, it is imperative to understand which pathogens, and in what prevalence, are carried by tick species in areas populated by humans. Few studies exist surveying the presence and distribution of tick-borne pathogens in the state of Delaware. The goal of this study was to create multiplex real-time PCR assays to identify *Borrelia burgdorferi sensu stricto, Babesia microti, Anaplasma phagocytophilum, Ehrlichia chaffeensis,* and *Ehrlichia ewingii* from their respective tick vectors collected across the state of Delaware.

Two multiplex, real-time PCR assays were developed and tested on 1027 ticks comprising *Ixodes scapularis* and *Amblyomma americanum*, two species of ticks commonly encountered in Delaware. The results showed that in a sample of 500 *Ixodes scapularis* ticks, 30.20 % were positive for *Borrelia burgdorferi*, 2.60 % were positive for *Babesia microti*, and 1 % were positive for *Anaplasma phagocytophilum*. Testing of 527 *A. americanum* ticks showed that 4.74 % were positive for *E. chaffeensis* and 1.14 % were positive for *E. ewingii*. These findings suggest that these five tick-borne pathogens are present across the state of Delaware and therefore pose a risk to the public.

## Highlights

• First statewide surveillance of tick-borne pathogens in Delaware.
• Two real-time PCR assays were developed to identify tick-borne pathogens in *Ixodes scapularis* and *Amblyomma americanum* ticks.
• Relative frequency of identify tick-borne pathogens found in ticks of Delaware was comparable to nearby states.

## 1 Introduction

*Ixodes scapularis* and *Amblyomma americanum* have been shown to vector several tick-borne pathogens that pose a risk to the public (Rochlin). Several studies have shown their prevalence across the eastern United States, as well as their ability to infect humans and other hosts with such tick-borne pathogens as *Borrelia burgdorferi sensu lato, Babesia microti, Anaplasma phagocytophilum, Ehrlichia chaffeensis* and *E. ewingii* (EisenEisen; Hojgaard et al., 2014; Kennedy and Marshall, 2021; Wright et al., 2014). However, little is known about the prevalence of these tick-borne pathogens in Delaware. Between 1998 and 2019, 430 ticks from Delaware were submitted to the Defense Centers for Public Health's Tick-Borne Disease Laboratory in Aberdeen Proving Grounds, Maryland for pathogen testing (Kennedy and Marshall, 2021). In a verified subset of these ticks, four (1.5 %) *Amblyomma americanum* ticks tested positive for pathogens; two were positive for *Ehrlichia ewingii*, one for *Ehrlichia chaffeensis*, and one *Ixodes scapularis* for *Borrelia* sp. In 2018, 258 *Ixodes scapularis* nymphs from New Castle County, Delaware were tested for *Borrelia burgdorferi, Borrelia miyamotoi, Anaplasma phagocytophilum,* and *Babesia microti* (Adalsteinsson). In the 258 Ixodes scapularis nymphs tested, twenty-nine ticks (11.2 %) tested positive for *Borrelia burgdorferi*, five ticks (1.9 %) tested positive for *Anaplasma phagocytophilum* and two ticks (0.8 %) tested positive for *Borrelia miyamotoi*. No ticks from this study were found to have been infected with *Babesia microti*. In 2019, 119 *Amblyomma maculatum* ticks collected from Delaware were tested for *Rickettsia parkeri* (Maestas). Of the 119 ticks studied, 25 (21 %) of the screened adult *A. maculatum* were found to be positive for *R. parkeri.* However, these few studies do not represent a comprehensive, geographic representation of the prevalence of *B. burgdorferi, Ba. microti, A. phagocytophilum, E. chaffeensis and E. ewingii* across the state of Delaware.

This study aimed to create a set of tests for each of these two tick species using the most up-to-date genetic information available, as well as deploying the tests on a sample of ticks collected from across the state of Delaware. One Multiplex assay, termed ISC1, was designed for *Ixodes scapularis* ticks to test for the following targets: trp-tRNA ligase (hypothetical protein) from gB31 genomic DNA for *Borrelia burgdorferi sensu stricto, 18s* rRNA for *Babesia microti* and *msp4* for *Anaplasma phagocytophilum.* The other multiplex assay, termed AAM1, was designed to test for, and distinguish between, *E. chaffeensis and E. ewingii* using the following targets: *120 omp* for *E. chaffeensis* and *omp/p28* for *E. ewingii*.

## 2 Materials and methods

*In silico* design testing was performed using the NCBI BLAST database, Primer Express 3.0.1 (Thermofisher, Waltham, MA), and Snapgene® (GSL Biotech LLC, Chicago, IL). The gene sequences for each target, as well as relevant homologs, were uploaded to Snapgene® software and submitted to a multiple DNA sequence MUSCLE alignment. The results of the alignment were used to verify unique and specific regions of the target sequences. After verification, the target DNA sequences were uploaded to Primer Express 3.0.1, a software designed to test sequences for use with QSY probes (Primer Express® Software v3.0.1, 2011). Primer and probe sequences chosen for downstream use in this study were based in part on the penalty score of the primer and probe sets in Primer Express software. To test in silico for species specificity and non-specific binding, a Basic Local Alignment Search Tool (BLASTn) using the National Center for Biotechnology Information (NCBI) website (https://blast.ncbi.nlm.nih.gov/Blast.cgi) was performed on all primer and probe sequences.

The exogenous controls were genomic DNA (gDNA) for each target organism (ATCC, BEI Resources, CDC) and cloned plasmids. The endogenous controls consisted of direct gDNA from pathogen-negative ticks spiked with gDNA (ATCC, BEI Resources, CDC) from each pathogen. Conventional PCR was performed on all endogenous and exogenous controls in 50 μl reactions using 25 μL of Promega GoTaq Green Master Mix®, .2 μM final concentration of each forward and reverse primer, 1 μL of pathogen or tick gDNA controls, normalized to 2 ng/μL, and sterile nuclease-free water to 50 μL. In addition, each target pathogen gDNA was tested not only with its intended primer sequences, but also with the primer sequences of the other four targets to test for cross reactivity. The Applied Biosystems MiniAmp Thermal Cycler (Thermo Scientific, Waltham, MA) was used for all standard PCR experiments. The Promega GoTaq Green Master Mix® thermal cycler protocol for these reactions was an initial denaturation at 95 °C for 2 min, followed by 35 cycles of 95 °C for 30 s, 60 °C for 30 s, and 72 °C for 1 min. Finally, a concluding extension of 72 °C for 5 min was performed. The 60 °C annealing temperature was used based on the stringent development of the primers to function best with this temperature. After PCR, 10 μL of each 50 μL sample was run on a 1 % agarose gel with 1 % Sodium Borate buffer and SybrSafe DNA stain (Thermofisher, Waltham, MA). Upon verification of a target amplicon via gel electrophoresis, the remaining 40 μL of the conventional PCR sample was cleaned the using the ReliaPrep™ DNA Clean-Up and Concentration System (Promega, Madison, WI) and were sequenced using the BigDye Terminator Cycle sequencing kit (Thermo Scientific, Waltham, MA).

After all PCR amplicon sequences were verified via Sanger Sequencing, positive control plasmids were created for downstream determination of the efficiency and the limit of detection (LOD) for both multiplex assays. Conventional PCR amplicons for each target were cloned into pGEM-T Easy Vector System I (Promega, Madison, WI) using the manufacturer's instructions for PCR ligation. After ligation, the plasmids were transformed into JM109 Competent *E. coli* cells plated on LB/amp/IPTG/X-gal plates and incubated at 37 °C overnight. Plasmids were isolated using the PureYield™ Plasmid miniprep System (Promega, Madison, WI) and sent to Plasmidsaurus™ (Eugene, OR) for full plasmid sequencing to ensure proper ligation of the target nucleotide sequence. Additional efficiency testing was performed using field-collected ticks that were positive for each pathogen, as well as testing pathogen-negative ticks, and pathogen-negative ticks spiked with pathogen gDNA. Table 1 represents the final primer and probe multiplex assay sequences for each target that performed the best.

**Table 1:** List of primers and probes developed in this study.

Pathogen	Locus	Genbank Accession #	Sequence 5’→3’ (Primer, Primer, Probe)
*B. burgdorferi*	Trp-tRNA Ligase (Hypothetical)	CP019767.1	CCCAACGGGAACTAAATTTGC
			GCAGCTGGACTTAAAGAGATTCCT
			FAM-CTCATAAGAACAGGATACCCCAAAAGGCCA-QSY
*Ba. microti*	18s rRNA	M93660.1	CGCGTGGCGTTTATTAGACTT
			GCCATGCGATTCGCTAATTT
			ABY-CCAACCCTTCGGGTAATCGGTGATTC-QSY
*A. phagocytophilum*	msp4	JQ522935.1	CAAGAATTTGAGCACGTTGAATG
			CGAACTTGAATGAGGGATCATG
			VIC-TTCAGATCCTGCCAGCTTCACGCA-QSY
*E. chaffeensis*	120 omp	AF474899.1	CGGAGTAATACTGATGGCTTGATAGA
			ACTGTGTCATCTTCTTGCTCTTGC
			FAM-TTGTGCAACCACAGGATCTGGGTTCTG-QSY
*E. ewingii*	omp/p28	AF287961.1	GATGCCCATATGTATTACCTTTTGG
			TGAGCAAGACAGATTTGTTAACTTGA
			JUN-TGTTGGCGAAGAACTATCAACTTCTCGTGC-QSY

The ticks were collected by the Delaware Department of Natural Resources and Environmental Control (DNREC) tick program from 24 locations across all three counties in the state of Delaware. Upon collection the ticks were submerged in 95 % ethanol and stored at -20 °C. The ticks were washed in ethanol followed by sterile water before dissection. Each tick was then bisected sagittally using a sterile scalpel. Half of each tick was placed in a storage tube and frozen at -80 °C. The other half of each tick was further dissected transversely and both transverse sections of each tick were placed in a labeled 1.5 mL microtube. DNA was isolated from 878 ticks using the tick supplementary protocol from the Qiagen DNeasy® Blood and Tissue Kit (Qiagen, Frederick, MD) and from the remaining 640 ticks using the Promega Maxwell 48 DNA isolation instrument (Madison, WI) with the Promega RSC Tissue DNA Isolation Kit (Madison, WI). Two DNA isolation methods were used because the Promega Maxwell 48 DNA isolation instrument was procured after assay development was in progress. The 260/280 and 260/230 purity values, as well as the concentration of the tick gDNA, were verified for each sample using the Nanodrop One Microvolume UV–Vis Spectrophotometer (Thermofisher, Waltham, MA).

After DNA isolation, the multiplex PCR assays of ISC1 and AAM1 were performed in 20 μL reactions containing 10 μL of Taqman Multiplex Master Mix™ (Thermofisher, Waltham, MA), a final concentration of 250 nM forward and reverse primers and probes for all targets relevant to the species of tick, 3 μL of pathogen gDNA, and nuclease-free water to 20 μL for each sample. The real-time standard curve protocol for testing was an initial 95 °C for 20 s, followed by 40 cycles of 95 °C for 1 s and 60 °C for 20 s on the QuantStudio 5™ Real-time PCR instrument (Thermofisher Scientific, Waltham. MA). Additionally, a *16s* gene block DNA sequence for *Babesia odocoilei* was tested in the multiplex to ensure there was no cross reactivity with the *Ba. microti* primers and probes.

To verify positive samples, each positive sample that resulted from the real-time PCR assay was subject to conventional PCR followed by Sanger sequencing verification. All the *Ba. microti, A. phagocytophilum, E. chaffeensis* and *E. ewingii* positives were subjected to conventional PCR and Sanger sequencing. Due to the relatively large number of *B. burgdorferi* positive samples, a random sample of 50 *B. burgdorferi* positive samples were chosen for verification. Subsequent sequence data was uploaded to Snapgene® software (San Diego, CA), and the sequences were verified via NCBI BLAST.

For all ticks, data was collected for the tick species, sex and developmental stage, date of collection, collection location and county, as well as the positive or negative tick-borne pathogen result. Positivity rates for each tick-borne pathogen were tabulated per the tick species, sex/developmental stage, and county. A Chi-Square test for homogeneity was performed with a significance level of α = 0.05 to identify any statistically significant differences in the positive percentages among ticks collected from the three counties in Delaware.

## 3 Results

The efficiencies of all the tested positive control plasmid targets were between 98 and 104 %, the average R^2^ values of the triplicate tests were between 0.9921 and 0.9994, and the slopes were between −3.15 and −3.65. LOD was also determined using the positive control plasmids from 10° to 10^−12^ in triplicate. The theoretical LOD for the five control plasmids ranged from 2 to 8 molecules. In addition, the efficiencies of all the pathogen-positive field-collected ticks as well as the pathogen-negative ticks spiked with pathogen gDNA were between 93 and 110 % with slopes between −3.2 and −3.6. To test the efficiency of the multiplex assays, an independent *t*-test with a significance level of 0.05 was used to compare the mean C_T_ values of each singleplex and multiplex trial. The smallest p-value for any singleplex versus multiplex trial was 0.789 and the largest p-value was 0.937, which signifies strong similarity in comparison of function.

The results of testing 500 *I. scapularis* ticks using the ISC1 Multiplex assay and the 527 *A. americanum* ticks using the AAM1 multiplex assay are listed in Table 2. In addition to the totals, there was evidence of coinfections of *I. scapularis* ticks with more than one pathogen. Of the 151 positive *B. burgdorferi* ticks, 7 ticks (4.6 %) were coinfected with *Ba. microti*. An additional 2 ticks (1.3 %) were infected with *A. phagocytophilum*. There was no evidence of *Ba. microti* positive ticks coinfected with *A. phagocytophilum*. There were no ticks that were coinfected with *B. burgdorferi*, *Ba. microti*, and *A. phagocytophilum*. The AAM1 multiplex showed evidence of one tick coinfected with *E. chaffeensis* and *E. ewingii*.

**Table 2:** Results of the percent positive ticks for tick-borne pathogens by County and life stage.

Tick-borne Pathogen (Tick species)	Overall Percent positive	Percent positive by county	Adult percent positive	Nymph percent positive
*B. burgdorferi (I. scapularis)*	(151/500) 30.2 %	NCC-(42) 23.73 %	(129/342) 37.72 %	(22/158) 13.92 %
		KC-(63) 37.95 %		
		SC- (46) 29.30 %		
*Ba. microti (I. scapularis)*	(13/500) 2.6 %	NCC-(4) 2.26 %	(8/342) 2.34 %	(5/158) 3.16 %
		KC-(0) 0 %		
		SC-(9) 5.73 %		
*A. phagocytophilum (I. scapularis)*	(5/500) 1 %	NCC-(3) 1.69 %	(2/342) 0.58 %	(3/158) 1.90 %
		KC-(1) 0.64 %		
		SC-(1) 0.64 %		
*E. chaffeensis (A. americanum)*	(25/527) 4.74 %	NCC-(2) 1.8 %	(22/157) 14.01 %	(3/370) 0.80 %
		KC-(0) 0.0 %		
		SC-(23) 6.23 %		
*E. ewingii (A. americanum)*	(6/527) 1.14 %	NCC-(0) 0.0 %	(6/157) 3.82 %	(0/370) 0.0 %
		KC-(0) 0.0 %		
		SC- (6) 1.63 %		

NCC= New Castle County KC= Kent County SC= Sussex County.

Geographic prevalence of the tick-borne pathogens tested from the 500 *I. scapularis* ticks revealed positive samples of *B. burgdorferi* and *A. phagocytophilum* from all three counties. Positive samples for *Ba. microti* were found in New Castle County (northernmost) and Sussex County (southernmost), but not in the central county of Kent County. Across the three counties, the prevalence of *B. burgdorferi* (χ = 8.31, p = 0.016), and *Ba. microti* (χ = 10.595, p = 0.050) were different, but the prevalence of *A. phagocytophilum* was not statistically different across the three counties (χ = 1.337, p = 0.512).

Testing of the 527 *A. americanum* ticks revealed positive samples of *E. chaffeensis* in both New Castle County and Sussex County, but not in Kent County. The prevalence of *E. chaffeensis* was different across the three counties (χ = 6.278, p = 0.043). Positive samples for *E. ewingii* were only found in Sussex County. The prevalence of *E. ewingii* was not statistically different across the three counties (χ = 2.599, p = 0.273).

## 4 Discussion

In relation to *A. phagocytophilum*, any positive *A. phagocytophilum* samples will need to be further sequenced to distinguish the V1 strain from the human disease-causing strains. The strain of *A. phagocytophilum* relevant to human disease is *A. phagocytophilum-human active (ha)*. This strain is known to infect humans and can be carried by myriad small rodents and wildlife, including the white-footed mouse (*Peromyscus leucopus*)(Keesing). The *A. phagocytophilum-ha* pathogen has been found in the *I. scapularis* tick and the *D*. *variabilis* tick, however vector competence has only been clearly established in *I. scapularis* (Holden et al., 2003). Another strain, *A. phagocytophilum-V1*, carried by wildlife, especially by white-tailed deer (*Odocoileus virginianus* Zimmermann), is not associated with human infection (Tate et al., 2005).

There exist published multiplex tests available for some of the same pathogens relevant to this study (Cohen et al., 2010; Graham et al., 2018; Hojgaard et al., 2014; Holden et al., 2003). Some of the tests that are still used today were developed a decade ago or more, when less genetic information was known about these pathogens than today. Furthermore, the assays for this study were developed using QSY quenchers, in contrast to BHQ1 or MGB. QSY quenchers are recommended for multiplexing because they come in a variety of dyes that support multiplexing. Because they are also nonfluorescent quenchers, real-time PCR instruments can measure the reporter dyes more precisely (Bonab). One drawback of QSY probes is that they normally require a longer sequence than other probe types such as MGB. This is due in part to the general requirement of QSY probe melting temperature of 70 °C. In addition, the primers used in Taqman QSY real-time PCR require primer sets to have a melting temperature, at, or close to 60 °C. These relatively stringent parameters sometimes make primer and probe development difficult. However, if sequences are found that are compatible, then specificity and sensitivity of these assays have been proven to be very effective for identification of intended target sequences (Bonab).

The efficiency testing of both multiplexes did not display any interference from the multiple primers and probes in the assays and successfully identified the intended pathogen DNA, as confirmed from subsequent sequencing of pathogen-positive ticks tested in this study. The results of the deployment of the ISC1 and AAM1 multiplex assays can provide evidence of the presence of tick-borne pathogens in ticks not only collected in Delaware, but anywhere else these pathogens may be found. Of interest, the percentage of pathogen-positive ticks tested in this study are similar to the percentages of findings in studies from nearby states. (Adelson et al., 2004; Cohen et al., 2010; Poje et al., 2022; Sakamoto et al., 2020; Wright et al., 2014). As more samples are tested, a more accurate representation of the frequency and distribution of these tick-borne pathogens in ticks of Delaware should be apparent. The creation and implementation of these real-time multiplex PCR assays for identifying the presence of tick-borne pathogens in Delaware is critical to providing stakeholders, researchers, and state agencies in Delaware with information relevant to public health.

## CRediT authorship contribution statement

**Michael H. Buoni:** Writing – review & editing, Writing – original draft, Methodology, Investigation, Funding acquisition, Conceptualization. **Ashley C. Kennedy:** Writing – review & editing, Funding acquisition. **Virginia Hughes:** Writing – review & editing. **Esther Biswas-Fiss:** Writing – review & editing.

## Declaration of competing interest

This letter officially states that I have no known conflicts of interest in the publication of this manuscript.
